# Anaerobic threshold using sweat lactate sensor under hypoxia

**DOI:** 10.1038/s41598-023-49369-7

**Published:** 2023-12-21

**Authors:** Hiroki Okawara, Yuji Iwasawa, Tomonori Sawada, Kazuhisa Sugai, Kyohei Daigo, Yuta Seki, Genki Ichihara, Daisuke Nakashima, Motoaki Sano, Masaya Nakamura, Kazuki Sato, Keiichi Fukuda, Yoshinori Katsumata

**Affiliations:** 1 Department of Orthopaedic Surgery, Keio University School of Medicine, Tokyo, Japan; 2 Department of Cardiology, Keio University School of Medicine, Tokyo, Japan; 3 Institute for Integrated Sports Medicine, Keio University School of Medicine, Tokyo, Japan

**Keywords:** Metabolism, Predictive markers

## Abstract

We aimed to investigate the reliability and validity of sweat lactate threshold (sLT) measurement based on the real-time monitoring of the transition in sweat lactate levels (sLA) under hypoxic exercise. In this cross-sectional study, 20 healthy participants who underwent exercise tests using respiratory gas analysis under hypoxia (fraction of inspired oxygen [FiO_2_], 15.4 ± 0.8%) in addition to normoxia (FiO_2_, 20.9%) were included; we simultaneously monitored sLA transition using a wearable lactate sensor. The initial significant elevation in sLA over the baseline was defined as sLT. Under hypoxia, real-time dynamic changes in sLA were successfully visualized, including a rapid, continual rise until volitionary exhaustion and a progressive reduction in the recovery phase. High intra- and inter-evaluator reliability was demonstrated for sLT’s repeat determinations (0.782 [0.607–0.898] and 0.933 [0.841–0.973]) as intraclass correlation coefficients [95% confidence interval]. sLT correlated with ventilatory threshold (VT) (r = 0.70, p < 0.01). A strong agreement was found in the Bland–Altman plot (mean difference/mean average time: − 15.5/550.8 s) under hypoxia. Our wearable device enabled continuous and real-time lactate assessment in sweat under hypoxic conditions in healthy participants with high reliability and validity, providing additional information to detect anaerobic thresholds in hypoxic conditions.

## Introduction

It is presumed that hypoxic training helps improve endurance performance in athletes^1^. Traditional high-altitude training refers to a state where atmospheric and oxygen pressure decrease and athletes are exposed to chronic hypobaric hypoxia for many weeks^2^. Recent studies on normobaric hypoxic exercise have investigated the impact of the recently popular live low-train high-altitude interventions on athletes’ lifestyles^3,4^, as prolonged exposure to low-pressure conditions is not always feasible (travel time, engagement, and expenses) and can lead to health problems^5,6^. Anaerobic threshold (AT) and peak oxygen uptake (peak VO_2_) should be routinely assessed in hypoxic conditions to practice efficient fitness training during hypoxia^7^. To date, the ventilatory threshold (VT), calculated as a noninvasive index of metabolic response to incremental exercise, has been used to determine AT^8,9^. The VT assessment method is beneficial; however, VT assessment requires an expensive analyzer and expertise due to the difficulty in confirming VT based on the oscillations in minute ventilation and inconsistencies among several factors^10^. The difference in expertise is reported to worsen the VT determination agreement^11^. This is because the respiratory gas analyzer is not readily available in sports settings. Therefore, there is an urgent need to apply an innovative and simple system to determine AT with high reliability for fitness training under hypoxia.

Flexible, wearable sensing devices can yield vital information about the underlying physiology of a human participant in a continuous, real-time, and noninvasive manner^12,13^. Sampling human sweat, rich in physiological information, can enable noninvasive monitoring^14^. We developed a sweat sensor to monitor sweat lactate levels (sLA) in real-time during progressive exercise in the clinical setting, investigating its use in detecting AT in healthy individuals and patients with cardiovascular diseases^15^. sLA has been reported to not reflect blood lactate during exercise^16,17^; however, our research group has examined sLA transitions during incremental load exercise and reported that the sweat lactate threshold was strongly approximated to AT by focusing on the inflection point where the value increases rapidly during incremental exercise, not the absolute value^15,18^.

Our sLA sensor is portable and easy to carry, enabling convenient measurements in various environments, and the continuous collection of only 1 Hz sLA values promises a simpler determination of the inflection point. Moreover, the need for invasive collection methods, including blood collection, is undesirable considering human resources for multi-measurements, the possibility of any person to evaluate, and acceptance of the evaluation target.

Under hypoxia, some researchers previously reported AT evaluation results^7^. However, similar to normoxia, it is problematic that the VT evaluation method was applied to broadly cover the sports setting. Therefore, we aimed to investigate the validity of AT estimation and reliability of the sLA continuously obtained using our sLA sensor during exercise under hypoxia in healthy participants.

## Results

The baseline characteristics of the healthy participants are summarized in Table 1. The participants were males (100%) with a median (IQR) age of 21 (20–21) years. The temperature and humidity were 28 ± 1 °C and 62 ± 6% under hypoxia, respectively.

**Table 1:** Baseline characteristics of participants.

Demographic and anthropometric data	Healthy male participants (n = 20)
Age (years)	21.0 (20.0–21.0)
Height (cm)	174.0 (171.0–176.4)
Body weight (kg)	69.0 (62.5–71.1)
BMI (kg/m^2^)	22.2 (20.5–23.4)
Body fat/body weight (%)	14.2 (12.3–16.7)
Body muscle/body weight (%)	81.3 (78.6–83.1)
Body water/body weight (%)	60.2 (57.5–62.8)

Data are presented as median (IQR). *BMI* body mass index.

**Figure 1:**
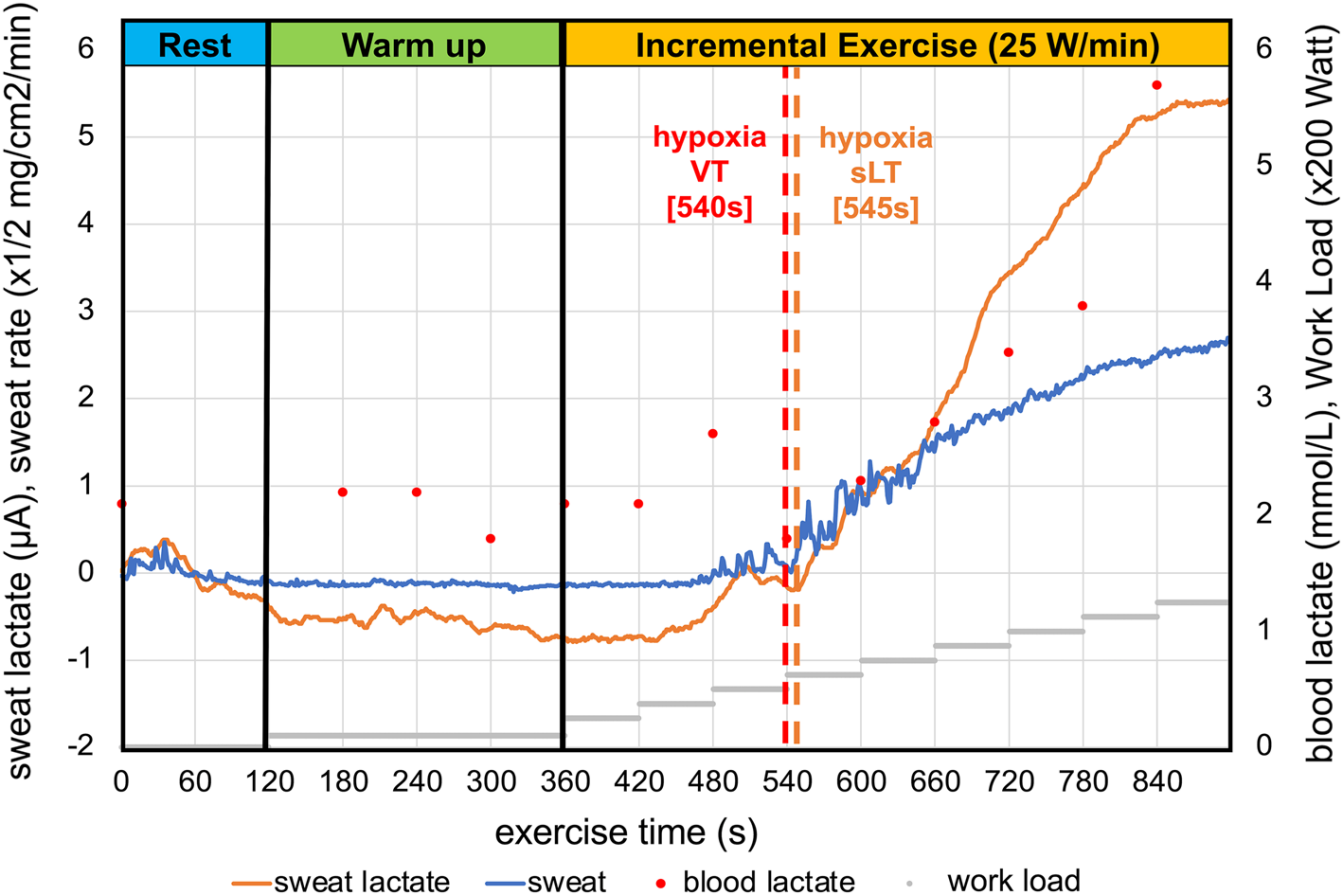
Imaging of sweat lactate levels, local sweat rate, and blood lactate values during incremental exercise under hypoxia. Representative graphs of sweat lactate levels (orange), local sweat rate (blue), and blood lactate values (red) during hypoxic exercise with a stepwise incremental protocol (25 W/min) ergometer are shown. *VT* ventilatory threshold, *sLT* sweat lactate threshold.

**Figure 2:**
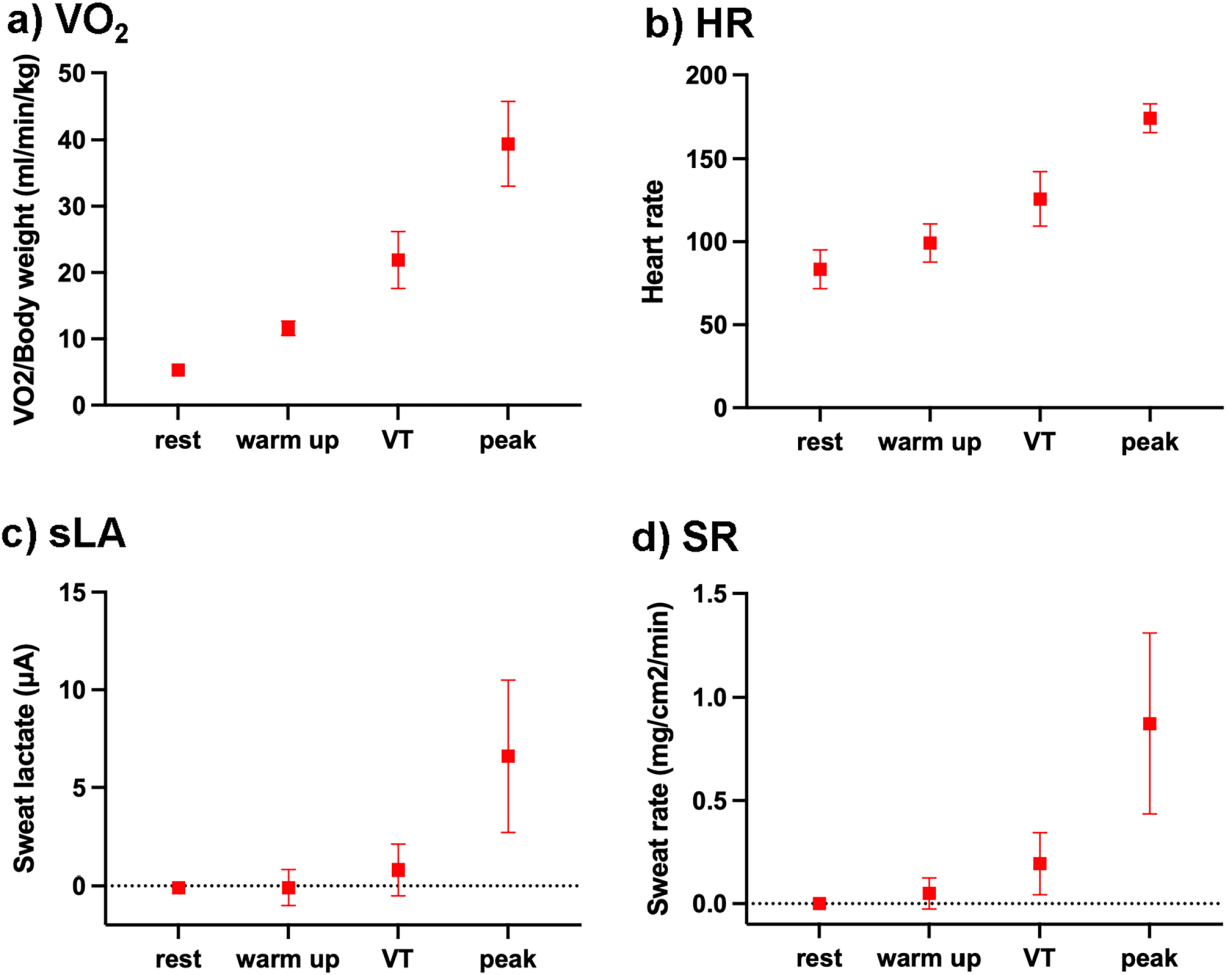
Measured parameters in hypoxia. The graph shows the measured parameters [(**a**) VO_2_/body weight, (**b**) Heart rate, (**c**) Sweat lactate, (**d**) sweat rate] at rest, warm up, VT, and peak in hypoxia. Data are shown as mean (± standard deviation). *VO*_*2*_ oxygen uptake, *VT* ventilatory threshold, *HR* heart rate, *sLA* sweat lactate, *SR* sweat rate.

Figures 1 and 2 show the sLA during exercise in hypoxia. During the exercise tests, dynamic changes in the sLA were continuously measured and projected onto the wearable device without delay, even under hypoxia. Because of the lack of sweat, the lactate biosensor measured a negligible current response at the commencement of cycling activity. During exercise, sLA increased drastically, and the sweat rate continuously increased as cycling continued until volitional exhaustion. This drastic sLA increase was not associated with the onset of sweating (Fig. 1).

Contrary to sLA, the heart rate and VO_2_ gradually increased from incremental-load exercise initiation to its end (Fig. 2). At the end of the exercise period, the sLA continued to decrease relatively slowly, mirroring the decrease in heart rate. The results under normoxia are shown in Supplementary Figs. 1 and 2.

**Figure 3:**
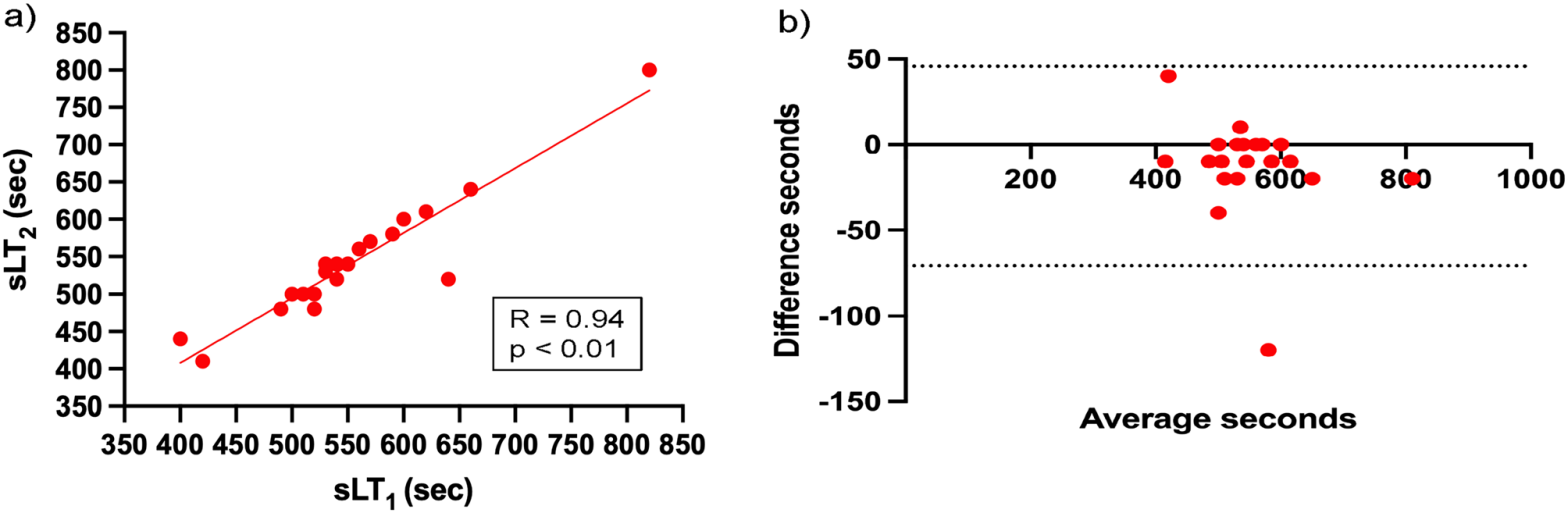
Reliability testing of the time at sLT determined by the same evaluator in hypoxia. (**a**) The graph shows the relationship between the repeatedly determined sweat lactate threshold (sLT) by the same evaluator. (**b**) The graph shows the Bland–Altman plots, which indicate the respective differences between the repeatedly determined sLT by the same evaluator (y-axis) for each individual against the mean of the time at the repeatedly determined sLT (x-axis) in hypoxia. *R* correlation coefficient, *p* p-value, *VT* ventilatory threshold, *sLT* sweat lactate threshold.

We easily identified the conversion from steady low lactate values to a continuous increase under hypoxia. Repeated sLT and VT determinations by the same evaluator demonstrated high intra-evaluator reliability (intraclass correlation [ICC] [2, 1] measured value [95% confidence interval] normo, 0.893 [0.794–0.952]; hypo, 0.782 [0.607–0.898]; and normo, 0.711 [0.500–0.861]; hypo, 0.919 [0.841–0.964]), respectively (Fig. 3 and Supplementary Fig. 3). Moreover, these were reproducible between both blinded reviewers (ICC [1, 1] measured value [95% confidence interval]; sLT; normo, 0.898 [0.765–0.958], hypo, 0.933 [0.841–0.973], and VT, normo, 0.933 [0.841–0.973], hypo, 0.836 [0.638–0.931]) as shown in Table 2 and Supplementary Table 1. However, the intra- and inter-evaluation reliability for bLT was low (ICC [1, 1] measured value [95% confidence interval]; normo, 0.529 [0.134–0.781], hypo, 0.652 [0.314–0.845], ICC [2, 1] measured value [95% confidence interval]; normo, 0.621 [0.363–0.813], hypo, 0.586 [0.331–0.790]) as shown in Fig. 3, Table 2, Supplementary Fig. 3, and Supplementary Table 1.

**Table 2:** Intra-evaluator reliability of sweat lactate threshold determination in hypoxia.

Hypoxia		N	Evaluator 1	Evaluator 2	Evaluator 3	ICC (95% CI)
sLT [s]	Mean	20	553.3	486.3	533.6	0.782 (0.607–0.898)
	SD		84.4	89.8	80.8	
bLT [s]	Mean	20	581.8	558.7	597.6	0.586 (0.331–0.790)
	SD		69.7	58.4	73.7	
VT [s]	Mean	20	546.8	547.2	540.9	0.919 (0.841–0.964)
	SD		66.2	49.8	59.7	

*ICC* intraclass correlation, *sLT* sweat lactate threshold, *bLT* blood lactate threshold, *VT* ventilatory threshold, *SD* standard deviation.

**Figure 4:**
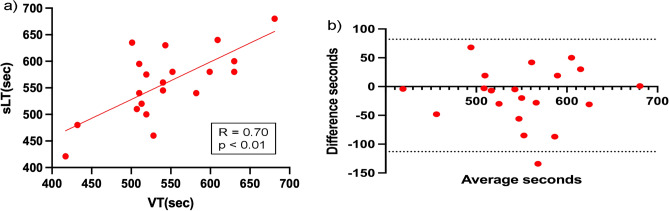
Validity testing of the time at VT and sLT in hypoxia. (**a**) The graph shows the relationship between the time from the start of the measurement (seconds) at VT and sLT. (**b**) The graph shows the Bland–Altman plots, which indicate the respective differences between the time from the start of measurement (s) at the VT and sLT (y-axis) for each individual against the mean of the time at the VT and sLT (x-axis) in hypoxia. *R* correlation coefficient, *VT* ventilatory threshold, *sLT* sweat lactate threshold.

The relationships between sLT and VT are shown in Fig. 4A and Supplementary Fig. 4A, describing the strong relationships between each threshold (normo, r = 0.69; hypo, r = 0.70). The Bland–Altman plot revealed that the mean difference between each threshold was 4.9 s under normoxia and − 15.5 s under hypoxia, and there was no bias between the mean values, displaying strong agreements between sLT and VT (Fig. 4B and Supplementary Fig. 4B).

## Discussion

**Figure 5:**
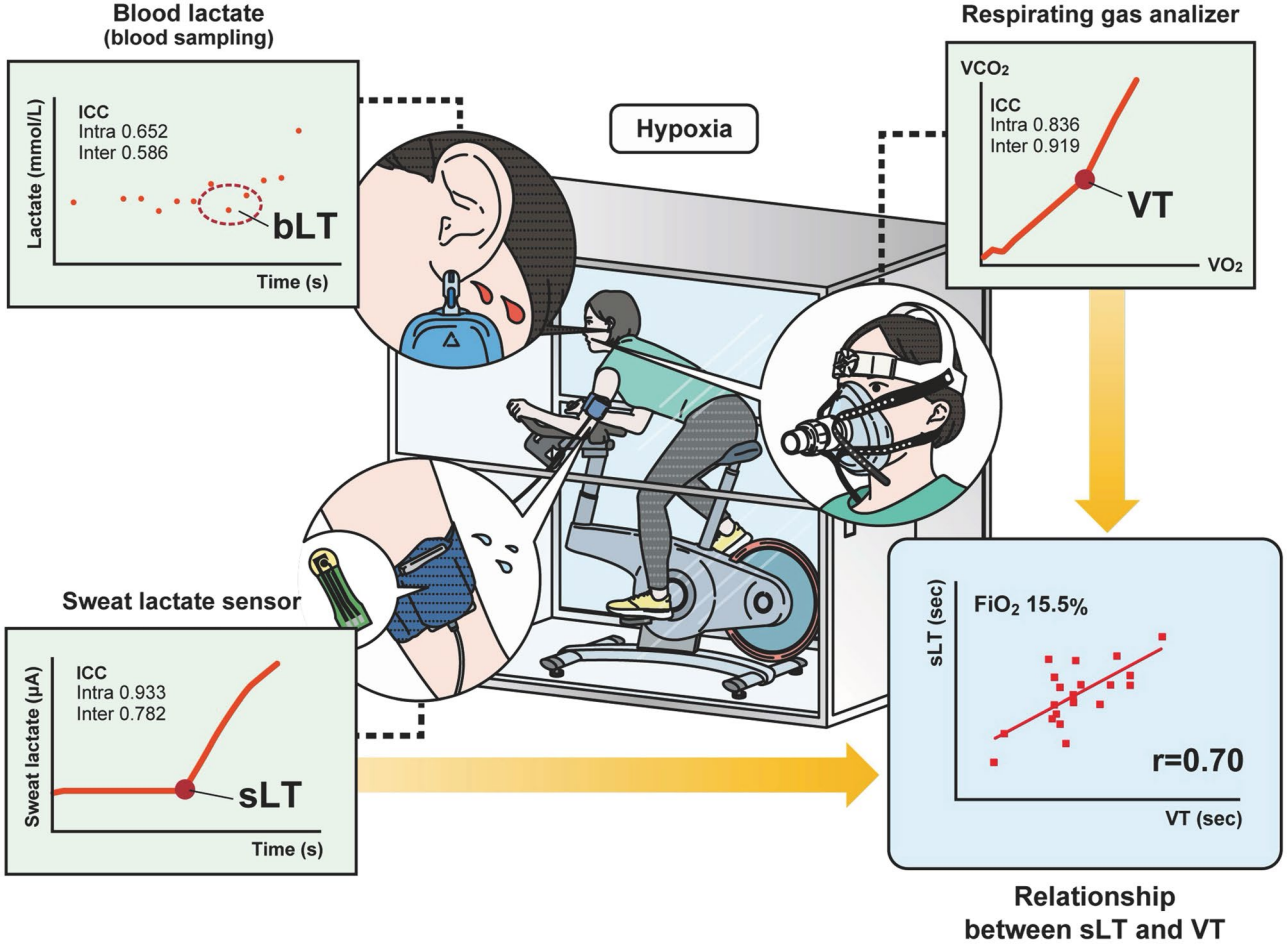
Schematic of the lactate-sensing device under hypoxia. This figure is licensed by © Medical FIG. *ICC* interclass correlation coefficients, *bLT* blood lactate threshold, *VCO*_*2*_ carbon dioxide output, *VO*_*2*_ oxygen uptake, *VT* ventilatory threshold, *sLT* sweat lactate threshold, *FiO*_*2*_ fraction of inspiratory oxygen.

The noninvasive sLA sensor enabled continuous and real-time measurement of sLA during an exercise test under hypoxia. Furthermore, sLT determination had high intra- and inter-evaluator reliability, and sLT was strongly correlated with VT. Real-time sweat lactate monitoring could be applied to detect aerobic threshold, even under hypoxia (Fig. 5).

Lactate levels are measured to track an individual’s performance and exertion level^19,20^. Blood lactate levels are measured by athletes or their supporters^21,22^, but these are not continuous, real-time measurements, limiting their utility to applications where stationary, infrequent tests are sufficient. In particular, applying bLT relies on the measurement’s reliability; in this study, the intra- and inter-evaluation reliability for bLT was low. Conversely, even under hypoxia, our devices captured the sLA during fitness in a real-time, noninvasive, and continuous manner at 1 Hz instead of cumulative values as in the conventional method, which detects the “timing of change” in a real-time and sensitive manner. Therefore, it is easy to identify the inflection point (sLT) from the plots of the sLA values. Using sLT demonstrated lower intra- and inter-observer bias and superior determination accuracy. Another possible explanation to support this positive result is that several operations, including the exchange of the sensor chip, cleaning the upper arm which the sensor fixed, and flushing out any residual sweat from the duct in the perspiration meter, certainly could eliminate the bias due to contaminations from previous experiments or original sweating. sLA has been reported to not reflect blood lactate during exercise^16,17^; however, our data showed that the AT point coincided with that in the sLA level during progressive exercise, consistent with the finding of the previous report^15,18^. This could be because an increase in lactate production from muscle cells, reflecting LT, may induce a simultaneous rise in sLA levels through changes in autonomic nervous balance, hormones, acid–base equilibrium, and metabolic dynamics^23,24^ similar to VT^25^. A previous study has demonstrated a rapid increase in blood catecholamine concentrations during incremental exercise loads^26^. Furthermore, it has also been indicated that sweat gland metabolism is activated by catecholamines^27^. Therefore, we are evaluating the timing of physiological responses to increasing exercise loads using completely different analytes and not estimating the bLA levels by observing the sweat lactate levels.

Measuring VT and peak VO_2_ with respiratory gas analysis helps in efficient training under hypoxia. However, it is often difficult to determine VT because of inconsistencies among the several factors required for detecting VT, such as the ventilation (VE)/oxygen uptake (VO_2_) or carbon dioxide production (VCO_2_)/VO_2_ slope and oscillations in minute ventilation^10^. Further, a respiratory gas analyzer is unavailable in a small hypoxic booth because of its size. Moreover, using a facemask, respiratory gas cannot be collected under hypoxic exercise. In addition, in a respiratory infection epidemic such as COVID-19, using respiratory gas analyzers has become difficult due to the possibility of cross-infection. Determining sLT using only sweat-based monitoring could overcome these problems, and the newly developed device enables AT measurements in various hypoxic environments (a small private booth and facemask).

It has been reported that sweat rate decreases in hypoxia^28^. As our sensor showed non-response in the absence of sweating, evaluating sweat rate is paramount to successfully determining sLT in hypoxia. This study quantified the amount of sweating per unit area near the sensor; the results showed no difference in the local sweat rate during exercise under hypoxia compared with that under normoxia. The relationship between the local sweat rate/response in the sLA sensor, humidity, and temperature during exercise warrants further investigation.

The device used in our study is suitable for use in remote monitoring or remote training settings during isolation measures, such as those taken during a respiratory infection epidemic. Furthermore, real-time assessments of sLA through a wireless data transfer system can offer a rigorous training menu under hypoxia based on the day-to-day physical conditions of trainees. In addition, exercise under hypoxia has been recognized as a new therapeutic modality for health promotion and disease prevention or treatment, such as for diabetes^29^, cardiovascular diseases^30^, hypertension^31^, obesity^32^, and age-related diseases^33^. Disease prevention and treatment can be more efficiently and safely provided by combining sLA sensors with exercise under hypoxia.

The study has some limitations. First, due to the observational study design, we could not exclude the influence of a selection bias. Second, our study had a relatively small number of cases. Third, the current study included healthy college-aged male individuals. Recent findings could be applied to various age groups and genders; however, further research, including females and young athletes, is required considering a sweat functional difference between sexes. Fourth, the sLA sensor used in this study exhibited the current value, not the sLA concentration. Conversion to concentration from the current value is possible; however, it is sufficient to display the current values to determine the inflection point based on the constant value of sLA during exercise. The effect of sLA dilution by high sweat rate on sLT determination is minimal due to the low sweat rate at AT and, therefore, does not negate our study’s result. Finally, exercise training has been performed under various hypoxic conditions; however, only a hypoxia of 15.5% was verified. Further verification is required to overcome these limitations.

In conclusion, the noninvasive sweat lactate sensor enabled continuous and real-time measurement of sweat lactate during exercise under hypoxia. The sweat lactate threshold can also be reliably determined by non-experts, even under hypoxia. Real-time sweat lactate monitoring could be used to detect aerobic threshold in a noninvasive and feasible manner under hypoxia and normoxia. It is expected that these findings enhance the effectiveness of exercise under hypoxia. This was the first study to show real-time monitoring of sLA during progressive exercise under hypoxia. Given the difficulty in deciding VT, such as in hypoxia, sLA monitoring could be beneficial in improving VT detection with high reliability.

## Methods

### Experimental approach to the problem

We conducted a cross-sectional study with 20 healthy participants who underwent exercise tests with respiratory gas analysis under hypoxia or normoxia and simultaneously monitored changes in sLA using a wearable lactate sensor to investigate the capability of sweat lactate sensor to monitor sLA under hypoxia and the relationship between sLT and VT. In addition, Intraclass correlation was determined for the intra- and inter-evaluator reliability of each threshold in this study.

### Subjects

Participants aged 20–80 years were recruited through a web system in June 2021. The exclusion criteria were patients receiving medication, having comorbidities like hypertension, diabetes, and active lung diseases, and having low local sweat rates of < 0.4 mg/cm^2^/min at the upper arm during maximal exercise. This sweat rate threshold was defined based on previous reports^15^ and preliminary studies. Twenty healthy participants were enrolled, including athletes and those with a broad spectrum of aerobic capacities and fitness levels. Notably, all participants exercised regularly for more than twice weekly.

The study protocol was approved by the Institutional Review Board (IRB) of Keio University School of Medicine (approval number 20190229), and the study was conducted following the principles of the Declaration of Helsinki. Verbal informed consent was obtained from all participants because the IRB approved using verbal consent following the Japanese guidelines for clinical research. Verbal consent was recorded as an experimental note.

### Procedures

The twice exercise tests with a minimum of 2 days intervals were performed using an electromagnetically braked ergometer (POWER MAX V3 Pro, Konami Sports Co., Ltd., Tokyo, Japan) with respiratory gas analysis under hypoxia (hypo; a fraction of inspired oxygen [FiO_2_], 15.4 ± 0.8% equivalent to a simulated altitude of 2500 m) or normoxia (normo; FiO_2_, 20.9%). Hypoxic conditions were created in an exercise booth with an oxygen filtration hypoxic generator (Hypoxico Everest Summit II; WILL Co., Tokyo, Japan) by insufflating nitrogen as a target of FiO_2_ 15.5%^34^. During exercise, the sLA was monitored using an sLA sensor (Grace Imaging Inc., Tokyo, Japan) attached to the upper arm, and the local sweat rate was measured at a sampling rate of 1 Hz in the same area as the sLA sensor using a perspiration meter (SKN-2000M; SKINOS Co., Ltd., Nagano, Japan). A perspiration meter ensured the value returned to zero before the new experiment by flushing out any residual sweat from the duct. Heart rate was monitored using Duranta (Zaiken, Tokyo, Japan), and blood lactate levels were measured using a standard enzymatic method on a lactate analyzer (Lactate Pro2^®^, ARKRAY, Kyoto, Japan).

On the day of the exercise test, the participants avoided any prior heavy physical activity. The participants performed the test upright on an electronically braked ergometer. Following a 2-min rest to stabilize the heart rate and respiratory condition, the participants performed a 4-min warm-up pedaling at 20 W. Then, they exercised at increasing intensity until they could no longer maintain the pedaling rate (volitional exhaustion). The resistance was increased in 25-W increments from 50-W at 1-min intervals. Once the exercise tests were terminated, the participants were instructed to stop pedaling and remain on the ergometer for 3 min.

The expired gas flow collected through the mask was measured using a breath-by-breath automated system (Aeromonitor^®^, Minato Medical Science Co., Ltd., Osaka, Japan). This system was subjected to a three-way calibration process involving a flow volume sensor, gas analyzer, and delay time calibration. The gas analyzer was calibrated under hypoxia using 8% O_2_, assuming a minimum oxygen concentration of 8% in exhaled air during hypoxic exercise. Respiratory gas exchange, including VE, VO_2_, and VCO_2_, was continuously monitored and measured using a 10-s average. VT was determined using the ventilatory equivalent, excess carbon dioxide, and modified V-slope methods^10^ through manual operating software. First, two of the three experienced researchers independently and randomly evaluated each participant’s VT using the three methods. The researchers used all three methods to assess concurrent breakpoints and eliminate false breakpoints. Second, if the VO_2_ values determined by the independent researchers were within 3%, then the VO_2_ values from the two investigators were averaged. Third, if the VO_2_ values determined by the independent evaluators were not within 3% of one another, a third researcher independently determined VO_2_. The third VO_2_ value was then compared with that obtained by the initial investigators. If the adjudicated VO_2_ value was within 3% of either of the initial investigators, the two VO_2_ values were averaged.

Blood lactate values were obtained by auricular pricking and gentle squeezing of the ear lobe to obtain a capillary blood sample at rest, warm-up, and every minute after the start of progressive intensity. The samples were immediately analyzed for whole-blood lactate concentrations (mmol/L).

bLT was determined through graphical plots of the bLA value vs. time^8^. Visual interpretation was independently made for each participant by two experienced researchers to locate the first rise from baseline. If the independent determinations of the stage at LT differed between the two researchers, a third researcher adjudicated the difference by independently determining LT. The three researchers then jointly agreed on the LT point.

The sLA was measured using a sLA sensor, which quantifies lactate concentration as a current value because it reacts with sLA and generates an electric current^15^. The sLA sensing system comprises a disposable sensor chip and a sensor. The sensor chip generates the current value proportional to the lactate concentration by catalyzing the enzymatic immobilization on its surface to oxidize lactate, which reduces hydrogen peroxide. In addition, a protective film formed by exposure using a UV lamp allows the achievement of immediate responsiveness (response delay < 1 s) and sustainability without the enzyme reacting all at once^15^. The current value can be obtained as continuous data within 0.1–80 μA in 0.1-μA increments. The sLA sensor responded linearly to the lactate concentrations, especially in the 0–5 mmol/L range, which were most significant in determining the LT because the LT had normal lactate values from 2 to 4 mmol/L^15^.

Moreover, it is also validated that the sLA values obtained from this sensor can show a significant enough difference to determine the inflection point under various sweat environments^35^. After calibration using saline for 2 or 3 min, the sensor chip connected to the sensor device was attached to the superior right upper limb of the participants and cleaned with an alcohol-free cloth to eliminate the influence of original sweat. In addition, the data were recorded at a sampling frequency of 1 Hz for mobile applications with Bluetooth connection. The recorded data were converted to moving average values over 13-s intervals and underwent zero correction using the baseline value. sLT was defined as the first significant increase in the sLA above baseline based on graphical plots^15^. Three researchers, independent of those who analyzed respiratory gas exchange, agreed on the point of sLT.

### Statistical analyses

The results are represented as mean ± standard deviation for continuous variables and percentages for categorical variables, as appropriate. ICC was determined for intra- and inter-evaluator reliability of each threshold^36^. The intra-evaluator reliability was tested by one of the blinded reviewers. The inter-observer reliability was tested by estimating each threshold using three blinded reviewers. The relationship between exercise time at sLT and VT was investigated using Pearson’s correlation coefficient test. In addition, the Bland–Altman technique was applied to verify the similarities among the different methods^37^. The graphical representation of the difference between the methods and the average WAS compared. Statistical significance was set at two-tailed p-values < 0.05. All statistical analyses were performed using IBM SPSS Statistics for Windows, version 27.0 (IBM Corporation, Armonk, NY, USA).

### Supplementary Information

Supplementary Information.

## Data Availability

The datasets used and/or analyzed during the current study are available from the corresponding author upon reasonable request.
