# Supplementary material for: Anaerobic threshold using sweat lactate sensor under hypoxia
Source: Sci Rep. 2023 Dec 21;13:22865. doi: 10.1038/s41598-023-49369-7 (PMC10739691; doi:10.1038/s41598-023-49369-7)
Supplement: Supplementary file 1 — Supplementary Information. [file 41598_2023_49369_MOESM1_ESM.pdf]

Supplementary Online Material

Anaerobic Threshold Using Sweat Lactate Sensor under Hypoxia

Hiroki Okawara, Yuji Iwasawa, Tomonori Sawada, Kazuhisa Sugai, Kyohei Daigo, Yuta Seki, Genki Ichihara, Daisuke Nakashima, Motoaki Sano, Masaya Nakamura, Kazuki Sato, Keiichi Fukuda, Yoshinori Katsumata

This supplementary material has been provided by the authors to give readers additional information about their work.

APPENDIX

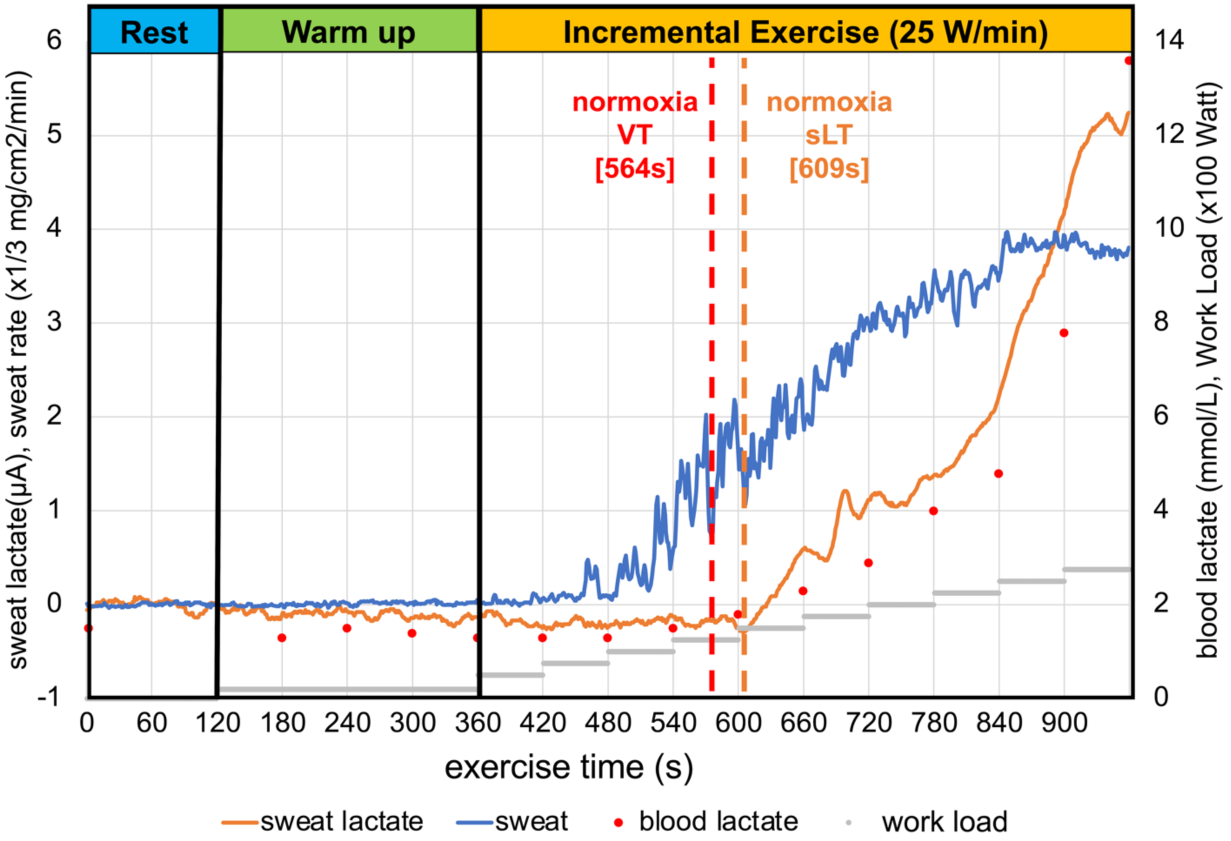

Supplementary Figure 1. Imaging of sweat lactate levels, local sweat rate, and blood lactate values during incremental exercise under normoxia

Representative graphs of sweat lactate levels (orange), local sweat rate (blue), and blood lactate values (red) during hypoxic exercise with a stepwise incremental protocol (25 W/min) ergometer are shown.

Abbreviations: VT=ventilatory threshold; sLT=sweat lactate threshold.

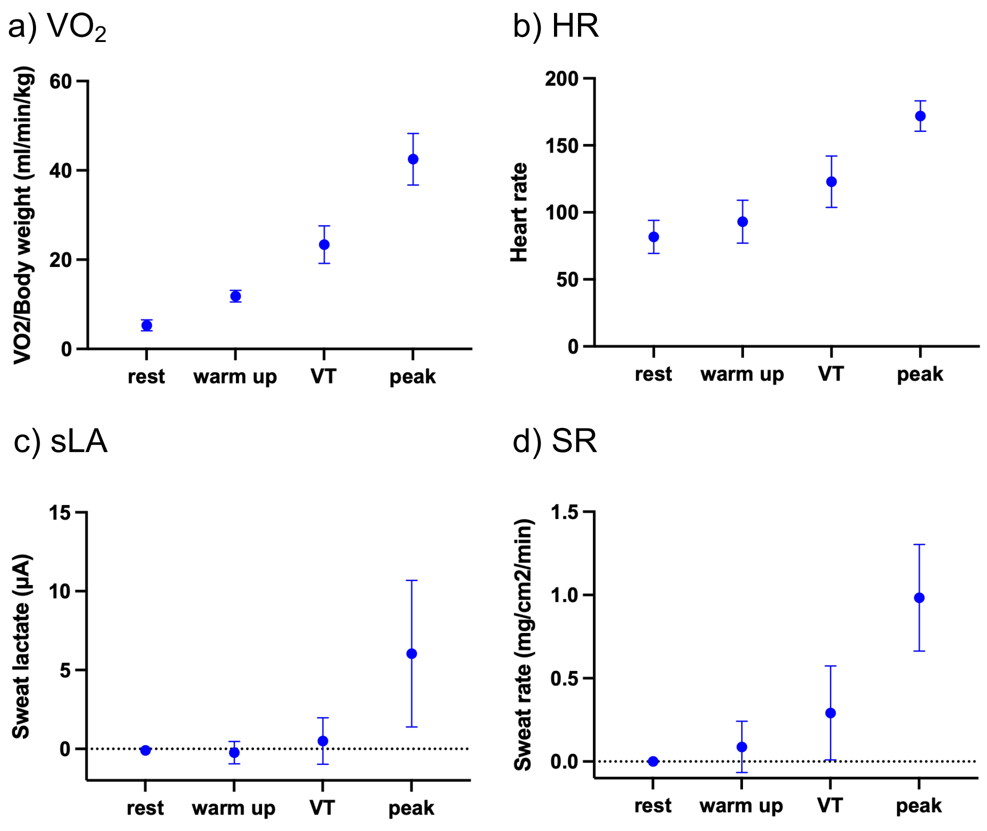

Supplementary Figure 2. Measured parameters in normoxia.

The graph shows the measured parameters (a; VO2/Body weight, b; Heart rate, c; Sweat lactate, d; sweat rate) at rest, warm up, VT, and peak in hypoxia. Data are shown as mean (±standard deviation).

Abbreviations: VO_2=oxygen uptake: VT=ventilatory threshold: HR=heart rate: sLA=sweat lactate: SR=sweat rate.

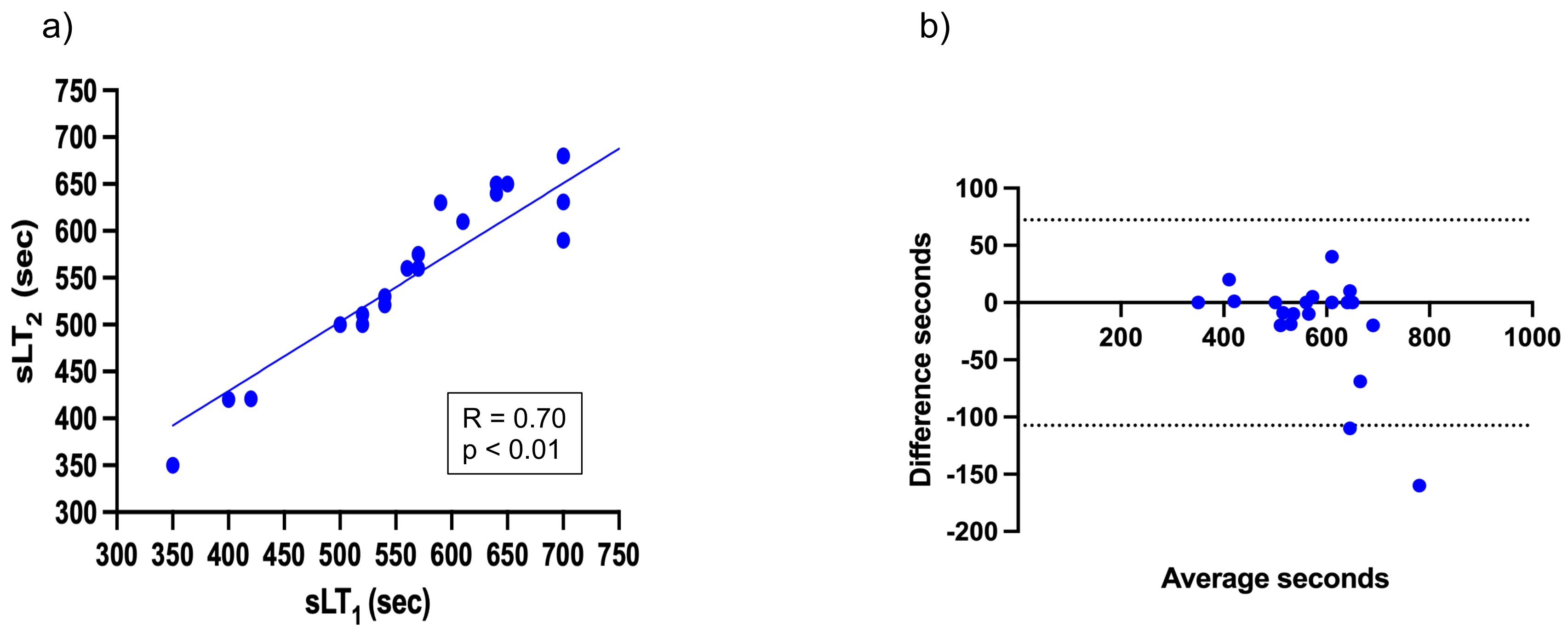

Supplementary Figure 3. Reliability testing of the time at sLT determined by the same evaluator in normoxia.

(a) The graph shows the relationship between the repeatedly determined sweat lactate threshold (sLT) by the same evaluator (b) The graph shows the Bland–Altman plots, which indicate the respective differences between the repeatedly determined sLT by the same evaluator (y-axis) for each individual against the mean of the time at the repeatedly determined sLT (x-axis) in normoxia. R, correlation coefficient; p, p-value; ventilatory threshold; sLT, sweat lactate threshold.

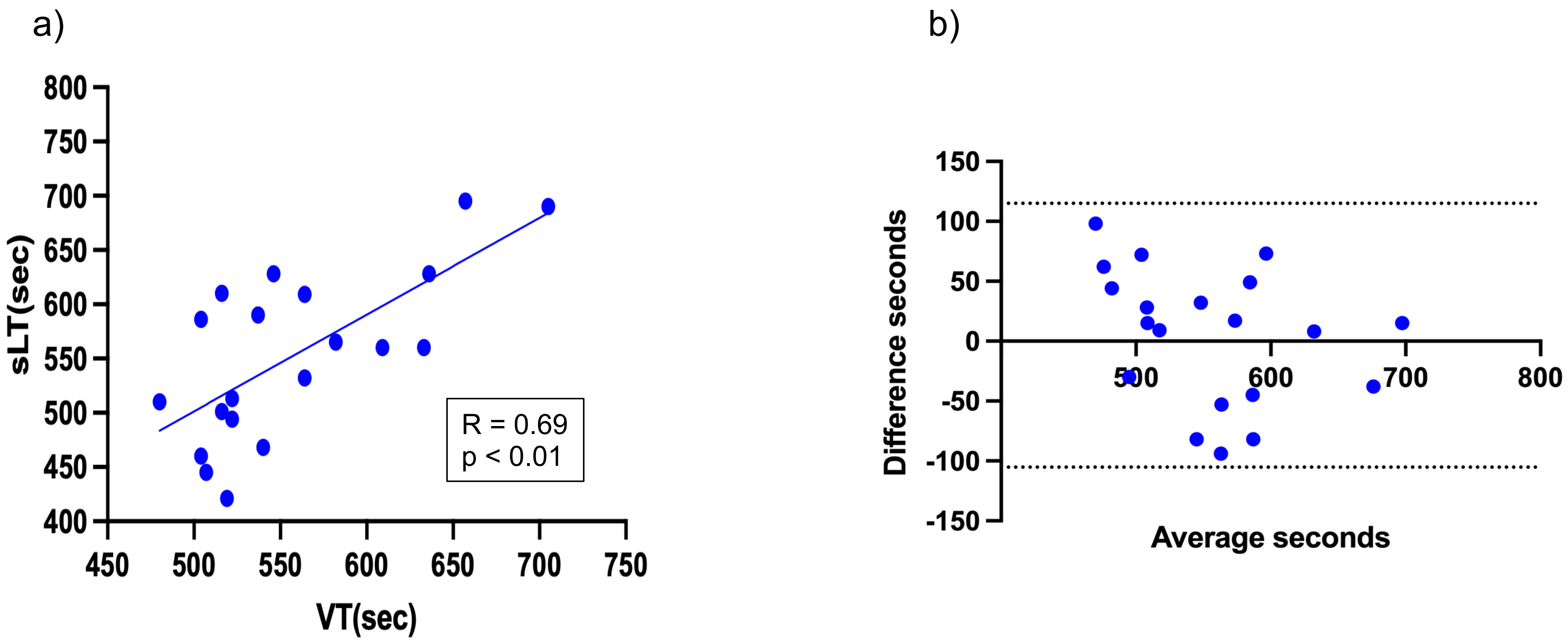

Supplementary Figure 4. Validity testing of the time at VT and sLT in normoxia

(a) The graph shows the relationship between the time from the start of the measurement (seconds) at VT and sLT. (b) The graph shows the Bland–Altman plots, which indicate the respective differences between the time from the start of measurement (s) at the VT and sLT (y-axis) for each individual against the mean of the time at the VT and sLT (x-axis) in hypoxia. R, correlation coefficient; VT, ventilatory threshold; sLT, sweat lactate threshold.

Supplementary Table 1. Intra-evaluator reliability of sweat lactate threshold determination in normoxia

Hypoxia		N	Evaluator 1	Evaluator 2	Evaluator 3	ICC (95%CI)
sLT [sec]	Mean	20	553.3	486.3	533.6	0.782 (0.607 - 0.898)
	SD		84.4	89.8	80.8	
bLT [sec]	Mean	20	643.9	605.6	611.3	0.621 (0.363 - 0.813)
	SD		77.1	67.4	81.9	
VT [sec]	Mean	20	563.1	552.2	552.6	0.711 (0.500 - 0.861)
	SD		56.5	45.3	60.0	

ICC, intraclass correlation; sLT, sweat lactate threshold; bLT, blood lactate threshold; VT, ventilatory threshold; SD, standard deviation.
